# *Orthopoxvirus* Antibodies in Feral Mammals in Mpox Outbreak Areas, Nigeria, 2021–2022

**DOI:** 10.3201/eid3205.251565

**Published:** 2026-05

**Authors:** Adeyinka Jeremy Adedeji, Clement Adebajo Meseko, Ismaila Ademola Shittu, Nanven Maurice, Suleiman Ladan, Emmanuel Obishakin, Dennis Kabantiyok, Rimfa Amos Gambo, Masdooq Aliyu, Odianosen Ehiakhamen, Ifeanyi Abali, Kwada Asunduwa Chagwa, Bitrus Vandi, Seyi Oyetunde, Nicodemus Mkpuma, Dorcas Gado, Besong Mathias Ayuk, Obadua Adegboyega, Akinbamidele Akinsorotan, Saleh Muhammad, Audrey Matheny, Clint N. Morgan, Yoshinori Nakazawa, Jeffrey B. Doty

**Affiliations:** National Veterinary Research Institute, Vom, Nigeria (A.J. Adedeji, C.A. Meseko, I.A. Shittu, N. Maurice, E. Obishakin, D. Kabantiyok, R.A. Gambo, M. Aliyu, S. Oyetunde, N. Mkpuma, D. Gado); Federal Ministry of Agriculture and Food Security, Abuja, Nigeria (S. Ladan, B.M. Ayuk); Nigeria Centre for Disease Control and Prevention, Abuja (O. Ehiakhamen, I. Abali); Adamawa State Ministry of Livestock and Aquaculture, Yola, Nigeria (K.A. Chagwa); Adamawa State Ministry of Environment, Yola (B. Vandi); Ondo State Ministry of Agriculture, Akure, Nigeria (O. Adegboyega, A. Akinsorotan); Nigeria Centre for Disease Control and Prevention, Abuja (S. Muhammad); US Centers for Disease Control and Prevention, Atlanta, Georgia, USA (A. Matheny, C.N. Morgan, Y. Nakazawa, J.B. Doty)

**Keywords:** *Orthopoxvirus*, viruses, antibodies, mpox, sylvatic, rodents, Nigeria

## Abstract

We analyzed tissue and serum samples from 124 wild animals from communities with confirmed mpox cases in Nigeria. Tissue samples were PCR-negative, but serum samples from 8 animals (6.45%)—3 feral cats, 4 giant pouched rats, and 1 shrew—revealed *Orthopoxvirus* antibodies, suggesting these species as probable reservoirs.

Members of the genus *Orthopoxvirus* are zoonotic pathogens belonging to the family Poxviridae (*1*–*3*). Orthopoxviruses (OPXVs) are capable of infecting a broad range of mammalian hosts via multiple routes, which could result in widespread infections and deaths in humans and animals (*1*,*2*). Monkeypox virus (MPXV) is a member of the genus *Orthopoxvirus* (*2*). OPXV infections are typically diagnosed by viral isolation and molecular assays, such as real-time PCR (*4*,*5*). Research has implicated rodents and small mammals as MPXV putative reservoirs (*6*–*9*). MPXV is endemic in animal reservoirs in West and Central Africa rainforest, and habitat encroachment and wildlife hunting are cited as likely factors associated with zoonotic spillover events (*8*,*10*). 

The first reported human mpox cases in Nigeria occurred in 1971 and then again in 1978; the disease reemerged in 2017 and has increased to endemic levels since then (*11*,*12*). During 2017–2025, the Nigeria Centre for Disease Control and Prevention (Abuja, Nigeria) reported 1,491 confirmed human cases of mpox in Nigeria, with 21 associated deaths. We conducted this study as part of a One Health investigation of the likely role of animal reservoirs in the transmission and maintenance of MPXV following reported human mpox cases in 2 states of Nigeria.

## The Study

**Figure 1:**
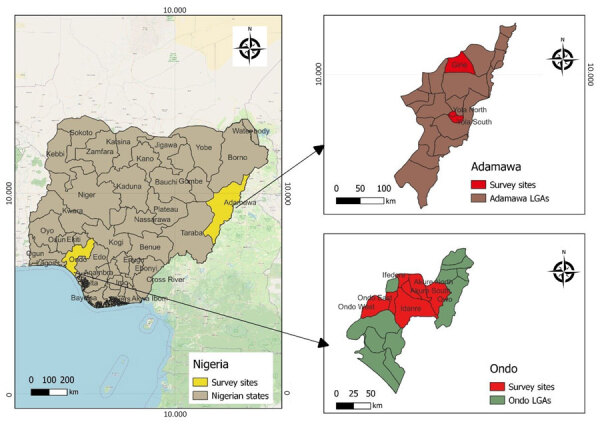
Sites of mpox animal surveillance activities for investigation of *Orthopoxvirus* antibodies in feral mammals in mpox outbreak areas, Adamawa and Ondo states, Nigeria, 2021–2022. LGA, Local Government Area.

In 2021–2022, Ondo State in Nigeria reported 40 confirmed human mpox cases (World Health Organization External Situation Report 8, October 2022, https://www.who.int/emergencies/situation-reports). Similarly, in 2022, Adamawa State (Figure 1), reported its index human mpox case in a serving soldier in Nigeria, followed by 28 mpox cases among inmates at a correctional facility in March 2022 (*13*). The correctional facility held inmates who were involved in terrorist activities and may have shared the same forest region with military personnel (*13*). Hence, in 2021–2022, health officials deployed a One Health mpox animal surveillance team (veterinarians, a physician, a microbiologist, and environmentalists) to investigate the role of animals in the upsurge of mpox cases in Ondo and Adamawa States.

Study locations in Ondo State included Idanre Forest Reserve, Owena Forest Reserve, Abule Olukete, Oba-Ile/Araromi, Oya, Emure, Igbara-Oke, and Akure metropolis (Figure 1). Our team also collected samples from wildlife purchased at bushmeat market stalls at Owena and Emure forest zones. Trapping sites in Adamawa State included a correctional facility, 2 public markets, and households with confirmed mpox cases (Figure 1). The animal surveillance team set Tomahawk live traps (https://tomahawklivetrap.org), Sherman live traps (https://shermantraps.com), and Victor snap traps (https://www.victorpest.com).

Before necropsy, we humanely euthanized trapped animals following standard operating procedures (*14*). We followed protocols approved by the National Veterinary Research Institute Animal Ethics Committee, Vom, Nigeria (AEC/03/53/18), and the US Centers for Disease Control and Prevention Institutional Animal Care and Use Committee, Atlanta, Georgia, USA (3183DOTMULX), in trapping and sampling rodents and small mammals. We collected blood samples by cardiac puncture and dropped samples onto Nobuto filter paper, allowing droplets to air dry before storing samples in a pouch with silica gel. We centrifuged the blood remaining in microtubes and aliquoted serum samples for storage in liquid nitrogen. We recorded age, species, and sex of each animal and took morphometric measurements as previously described to aid phenotypic species identification (*14*). We also examined each animal for pox-like lesions. We shipped the samples, collected in duplicate, to the National Veterinary Research Institute and the US Centers for Disease Control and Prevention for analysis.

We extracted DNA from tissue samples (liver, spleen, lungs, kidney, skin) collected from all 124 animals by using a MagMAX magnetic processor (Thermo Fisher Scientific, https://www.thermofisher.com) and conducted real-time PCR on all available samples by using OPXV-generic and MPXV-specific assays as previously described (*5*,*7*). To detect the presence of OPXV IgG, we tested all serum samples and dried blood spots using an in-house–developed ELISA, used in previous studies, at dilutions of 1:100, 1:200, and 1:400 (*6*–*8*). We used Western reserve vaccinia virus as the antigen to coat the plates (*7*). We considered serum samples that were reactive at 1:100 and 1:200 dilutions to be positive (*7*).

**Figure 2:**
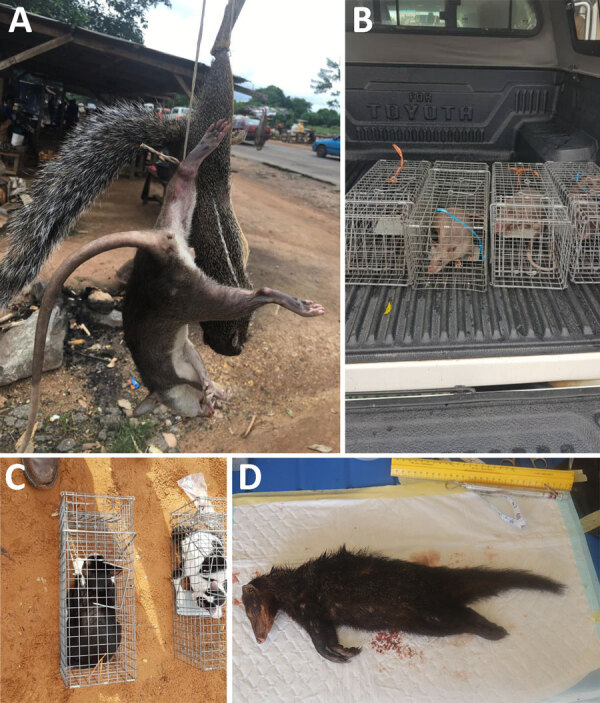
Animals collected for an investigation of *Orthopoxvirus* antibodies in feral mammals in mpox outbreak areas, Nigeria, 2021–2022. A) Freshly hunted giant pouched rat and stripped ground squirrel at bushmeat market in Ondo State. B) Trapped giant pouched rat in Yola in northern Adamawa state. C) Feral cats that inadvertently entered a Tomahawk live trap. D) Marsh mongoose bought at a wildlife market in Idanre Ondo state.

The survey team trapped 124 rodents and small mammals (Table). The identified species consisted of house mice (*Mus* spp.), black rats (*Rattus rattus*), white-toothed shrews (*Crocidura spp.*), giant pouched rats (*Cricetomys* spp.), and feral cats that were inadvertently caught in the live traps (Table; Figure 2). We also collected tissue samples (liver, spleen, lungs, skin) from 5 species of freshly hunted wild rodents and small mammals at bushmeat markets (Table, Figure 2). We observed no pox-like lesions on animals sampled.

**Table:** Geographic distribution of animals trapped or purchased from bushmeat markets in states of Ondo and Adamawa in study of *Orthopoxvirus* antibodies in feral mammals in mpox outbreak areas, Nigeria, 2021–2022

Scientific name	Common name	Adamawa	Ondo	Total
Animals trapped				
Feral cat	Cat	6	0	6
* Crocidura spp.*	Shrew	19	12	31
* Rattus rattus*	Black rat	21	0	21
* Mus spp.*	House mouse	6	5	11
* Cricetomys*	Giant pouched rat	50	5	55
Subtotal		102	22	124
Animals harvested from bushmeat markets				
* Dendrohyrax*	Tree hyrax	0	1	1
* Xerus erythropus*	Stripped ground squirrel	0	13	12
* Atilax paludinosus*	Marsh mongoose	0	1	1
* Atherurus africanus*	Brush-tailed porcupine	0	6	6
* Thryonomys swinderianus*	Marsh cane rat; grasscutter	0	30	30
Subtotal		0	50	50
Total		102	72	174

Laboratory results revealed no OPXV or MPXV DNA in tissue samples collected from the 174 rodents and other small mammals we studied. All blood samples on the Nobuto dry spots tested negative as well. However, we detected OPXV IgG in 8 (6.45%) of 124 serum samples collected from the trapped animals, specifically feral cats (3/6 [50%]), giant pouched rats, (4/55 [7.27%]), and a shrew (1/31 [3.23%]). All detected OPXV antibodies were in animals trapped in 2 local government areas in Adamawa State: Girei (shrew) and Yola North (feral cats, giant pouched rat), where confirmed human mpox cases were recently reported.

## Conclusion

In this study, we detected OPXV IgG in serum samples collected in 3 animal species in communities where human mpox cases were confirmed, suggesting that MPXV or other OPXVs may be circulating in animal populations. In our previous study, we found OPXV antibodies in rodents, namely *Praomys* spp. rodents and *R. rattus* rats, in a community with confirmed human mpox cases in Nigeria, further validating the hypothesis that rodents may be likely reservoirs of OPXV, such as MPXV or taterapox virus (*6*,*7*). We also detected OPXV antibodies in *Cricetomys* spp. rats, *Crocidura* spp. shrews, and feral or stray domestic cats. Other studies have demonstrated the presence of OPXV antibodies in *Cricetomys* spp. rats and *Crocidura* spp. shrews in some countries in African (*6*,*15*). Although our findings do not definitively confirm *Cricetomys* spp. rats or *Crocidura* spp. shrews as a sylvatic reservoir for OPVX or MPXV, this research does provide additional evidence of the possible role of those species in maintaining the viruses within the ecology. Of interest, the only positive sample associated with *Crocidura* spp. shrews was from an animal trapped in a household with no prior history of mpox. However, all locations in this study had reported nearby human mpox cases. 

It is possible that the IgG-positive animals we reported were exposed to OPXV or MPXV via human-to-animal transmission or reverse spillover. The proportion of stray cats in the study with OPXV antibodies was high (50%). All sampled stray cats were inadvertently captured at sites near confirmed human mpox cases. It is reasonable to hypothesize that the antibodies in those cats may have come from consuming potentially OPXV-infected rodents or from exposure to contaminated environments. A major limitation of this study was the inability of the serologic assay used to differentiate the OPXVs. It is likely that the animals may have been exposed to MPXV, but we cannot completely rule out the possibility of exposures to other OPXVs, including taterapox virus or an unidentified OPXV.

In summary, our study demonstrates the presence of OPXV antibodies in *Cricetomys* spp. rats, *Crocidura* spp. shrews, and feral cats in communities with confirmed human mpox cases in Nigeria. Our research offers further evidence of the possible role of small mammals as likely hosts of zoonotic OPXVs in nature.
